# Detecting and Tracking the Positions of Wild Ungulates Using Sound Recordings

**DOI:** 10.3390/s21030866

**Published:** 2021-01-28

**Authors:** Salem Ibrahim Salem, Kazuhiko Fujisao, Masayasu Maki, Tadanobu Okumura, Kazuo Oki

**Affiliations:** 1 Faculty of Engineering, Kyoto University of Advanced Science, 18 Yamanouchi Gotanda-cho, Ukyo-ku, Kyoto 615-8577, Japan; kazu@iis.u-tokyo.ac.jp; 2 Faculty of Engineering, Alexandria University, Lotfy El-Sied St. off Gamal Abd El-Naser-Alexandria, Ash Shatebi, Alexandria Governorate 11432, Egypt; 3 Institute of Industrial Science, The University of Tokyo, 4-6-1 Komaba, Meguro-ku, Tokyo 153-8505, Japan; fujisaok222@affrc.go.jp; 4 National Agriculture and Food Research Organization, 3-1-1 Kannondai, Tsukuba, Ibaraki 305-8517, Japan; 5 Faculty of Food and Agricultural Sciences, Fukushima University, 1 Kanayagawa, Fukushima 960-1296, Japan; makimasa@agri.fukushima-u.ac.jp; 6 Wildlife Management Office Inc., 922-7 Komiya-cho, Hachiouji, Tokyo 192-0031, Japan; okumura@wmo.co.jp

**Keywords:** wild ungulates, animals count, tracking animals, deer, Oze, sound recognition, repetitive calls, time drift, sound localization

## Abstract

Monitoring wild ungulates such as deer is a highly challenging issue faced by wildlife managers. Wild ungulates are increasing in number worldwide, causing damage to ecosystems. For effective management, the precise estimation of their population size and habitat is essential. Conventional methods used to estimate the population density of wild ungulates, such as the light census survey, are time-consuming with low accuracy and difficult to implement in harsh environments like muddy wetlands. On the other hand, unmanned aerial vehicles are difficult to use in areas with dense tree cover. Although the passive acoustic monitoring of animal sounds is commonly used to evaluate their diversity, the potential for detecting animal positions from their sound has not been sufficiently investigated. This study introduces a new technique for detecting and tracking deer position in the wild using sound recordings. The technique relies on the time lag among three recorders to estimate the position. A sound recording system was also developed to overcome the time drift problem in the internal clock of recorders, by receiving time information from GPS satellites. Determining deer position enables the elimination of repetitive calls from the same deer, thus providing a promising tool to track deer movement. The validation results revealed that the proposed technique can provide reasonable accuracy for the experimental and natural environment. The identification of deer calls in Oze National Park over a period of two hours emphasizes the great potential of the proposed technique to detect repetitive deer calls, and track deer movement. Hence, the technique is the first step toward designing an automated system for estimating the population of deer or other vocal animals using sound recordings.

## 1. Introduction

Over the past few decades, deer populations have been significantly growing along with an apparent expansion of their area of distribution in some regions of Europe [1], North America [2], and Japan [3], causing massive damage to farming and forestry [4]. Deer have also invaded many protected areas such as the Oze marshland [5], and Kushiro-shitsugen National Park [6] in Japan, resulting in irreversible effects on the original ecosystem such as destruction of rare plant species [7]. Monitoring the population dynamics of deer is thus crucial for the effective evaluation and management of ecosystems [8].

One of the methods used commonly to count the number of deer is the spotlight-census survey [1,9]; a light-census survey is a traditional technique using artificial light during nighttime to count the number of deer. Nevertheless, the light-census survey is strongly influenced by weather conditions, deer movements, and accessibility of the location, which causes considerable uncertainty in the observed data. Camera traps that are automatically triggered by movement have also been used to monitor the density of various wildlife animals such as American marten (*Martes americana*) [10], livestock [11], and wild ungulates [12,13] to evaluate their impact on the surrounding environment [14,15]. Thermal cameras have been also used to automatically detect animals in a grassland habitat [16]. The camera’s accuracy relies on the photographing frequency and the animal’s moving speed. Several other methods rely on walking around the survey region to search for deer and its sign, such as pellet counts [17,18] and quadrat methods [19,20]. However, these methods are not suitable for harsh environments (e.g., muddy wetlands) and some animals do not leave indices that can be counted [21,22]. Aerial surveys were used to survey marsh and pampas deer in Brazil during the dry season [23]. In recent years, the application of unmanned aerial vehicles (UAVs) for wildlife monitoring has expanded rapidly [24,25,26]. However, it is very difficult to use both aerial surveys and UAVs over areas with dense tree coverage, which obscure the detection of animals in both visible and infrared bands.

Sound recordings have been used by researchers to estimate animal density, such as counting yellow rails using autonomous recording units [27]. Sound-based techniques are suitable for rugged and difficult-to-reach areas (e.g., muddy or mountainous regions). Sound recordings have also been used to image the phonotactic behavior of female frogs in the dark [28]. Enari et al. [2,8] used passive acoustic monitoring to monitor sika deer in eastern Japan; they built a sound recognizer to automatically detect three types of deer calls from sound recordings. Their results showed that the fully automated procedure results in numerous false detections, and manual screening is needed to correct this issue. Passive acoustic telemetry techniques have been used to track the movement patterns of fishes and marine organisms [29]. The technique uses sound waves emitted from a tagged transmitter implanted in fishes in order to track them. The need to mark and recapture the fish or animals limits the application of acoustic telemetry techniques [2,30], particularly in open habitats where deer or other vocal animals live. Another limitation related to the sound-based technique is that repetitive calls from the same vocal animal must be identified and excluded during the counting process.

This study introduces a new sound-based technique to estimate deer’s position based on sound recordings of deer calls at three stationary recorders. The aim of the proposed research was to: (1) introduce a new technique to detect deer positions from their calls, and to identify repetitive calls of the same deer; (2) validate the technique using measurements from both controlled experiments and wild deer environments; and (3) discuss the strengths, limitations, and offer further suggestions for the proposed technique to be implemented for deer surveys.

## 2. Methods

### 2.1. Study Area

Two validation experiments (i.e., in the control environment, and in the natural environment of deer) were performed to provide a reliable validation of the proposed technique. During the two experiments, a deer whistle was used to emit deer calls at different locations (i.e., validation points), and that emitted sound was recorded using sound recorders installed in each experiment’s site (details in Section 3.1 and Section 3.2). Deer whistles have been effectively used to mimic, call, and hunt deer for many years [31,32].

**Figure 1:**
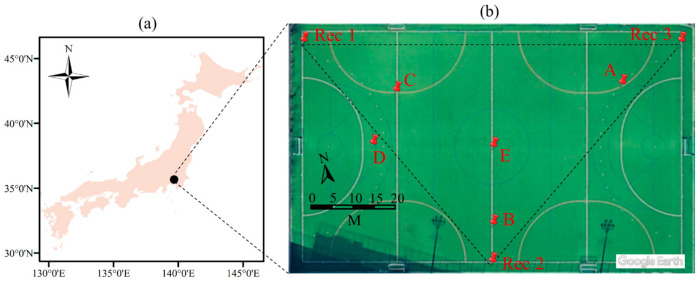
(**a**) First validation experiment site conducted on 29 November 2017 at University of Tokyo, Japan, and (**b**) location of the three recorders (i.e., Rec 1–3) and validation points (i.e., A–E) where deer calls were emitted using a deer whistle. Playground image from Google Earth.

The first validation experiment was conducted at a playground located in the University of Tokyo, Japan (hereafter called UTokyo experiment), with 90 m length and 55 m width, as shown in Figure 1b. Three recorders were installed and pointed toward the center of the playground (Figure 1b). Five validation points (A–E, as shown in Figure 1b) were selected to validate the proposed technique. At each validation point, deer calls were emitted using deer whistle and recorded by the three recorders. The coordinates of the three recorders and five validation points are summarized in Table 1.

**Figure 2:**
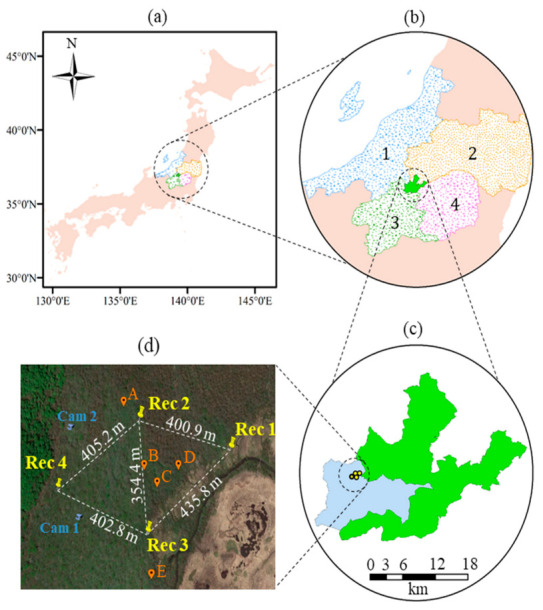
Location of recorders installed in Oze marshland. (**a**) Location of Oze National Park in central Japan; (**b**) location of Oze National Park with respect to four Japanese prefectures (i.e., 1-Niigata, 2-Fukushima, 3-Gunma and 4-Tochigi); (**c**) special protected area of Oze National Park (in blue); and (**d**) Google Earth image indicating location of recorders (Rec 1–4), distance between recorders, locations of two camera traps (Cam 1 and 2) and locations of validation points (A–E) where deer calls were emitted using a deer whistle.

The second validation experiment was conducted in Oze National Park (Figure 2). Oze National Park is located in central Japan (Figure 2a), with an area of 372 km^2^ [33]. It spans four Japanese prefectures (Niigata, Fukushima, Gunma, and Tochigi prefectures), as shown in Figure 2b. Oze is famous for its rare plant species, and a wide variety of wildlife [34]. Recently, there has been a significant increase in deer population in Oze, causing irreversible damage to rare plants. Like the UTokyo experiment, the proposed technique was also validated in Oze National Park at five points (A–E, as shown in Figure 2d) using a deer whistle. Four sound recorders were installed in Oze Marshland (Figure 2d), which located in the special protected area (Figure 2c) of Oze National Park. The coordinates of the four sound recorders and five validation points are listed in Table 1. The real deer calls were also collected in Oze site during the rutting season (i.e., October 2018). The data of two camera traps (Ltl Acorn 6310W; LTL Acorn Outdoors, Green Bay, WI, USA) located within the Oze site were used to validate the retrieved deer positions from deer calls (Section 3.3). The Ltl-6310w camera is equipped with a wide field of view of 100° and a passive infra-red (PIR) sensor to detect the sudden change of ambient temperature caused by a moving object. The two cameras were set to capture an image and then record a video for 20 s once the PIR sensor captures a motion. Shapefiles used to create Figure 2 were downloaded from the Japanese National Land Numerical Information database [35].

### 2.2. Description of Proposed Technique

Sounds provide valuable information about the deer producing them, such as sex and age (buck, doe or fawn), emotion (e.g., snort sound during danger or estrus bleat that is made to attract bucks) [31], and rough information about their position. The proposed technique aims to determine the accurate position of deer (i.e., latitude and longitude of deer position) from their sound recordings. By knowing the precise location of deer, we can identify repetitive calls from the same deer and avoid counting them, as well as track its movement. Our technique relies on using three synchronous stationary recorders to record deer calls.

**Figure 3:**
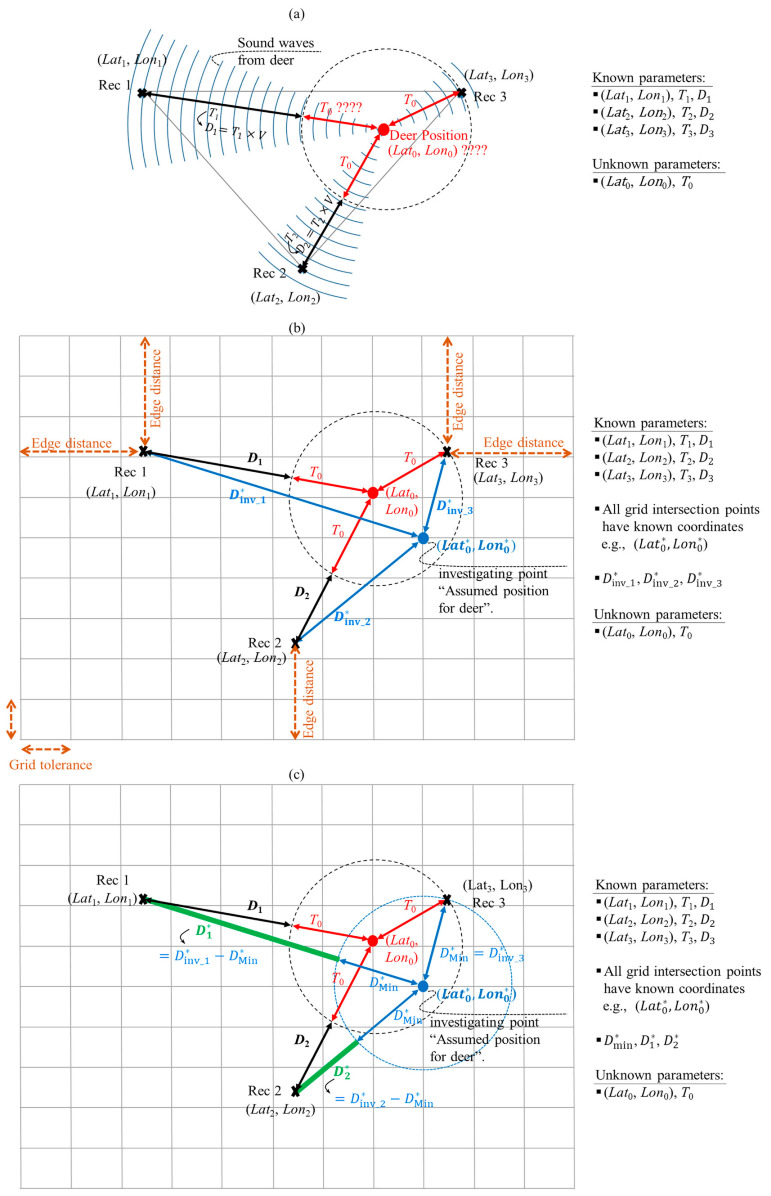
Schematic diagrams explaining the concept of the proposed technique. (**a**) Available and unknown information based on the sound recorders’ positions and sound recordings, (**b**) trial-and-error method to retrieve deer position where the blue point represents the investigated point and the grid’s intersection points represent the candidate positions of deer, and (**c**) distances corresponding to the time lag of deer call ($D_{1}$, $D_{2}$ ) and investigated point ($D_{1}^{*},\;D_{2}^{*}$ ).

The concept of detecting deer position using the three sound recordings is as follows. When a deer makes a sound, the sound waves propagate in a series of spherical waves in all directions and are recorded at the three stationary recorders at times that are based on the location of the recorders with respect to the position of the deer. From these times, the time lag (i.e., time-delay) to detect the deer sound among the three recorders can be estimated. The position of deer can be retrieved from these time lags. As shown in Figure 3, the three recorders (i.e., Rec 1–3) have known positions (i.e., $Lat_{x},\;Lon_{x}$; where x = 1, 2, and 3), whereas the deer position (i.e., $Lat_{0},\;Lon_{0}$) is unknown. Rec 3 is the closest to the deer position, whereas Rec 1 is the farthest. Thus, the deer sound will be first detected by Rec 3, then Rec 2, and then Rec 1. Of course, the time duration (*T*_0_) that the deer sound requires to reach the nearest recorder from the deer position (i.e., Rec 3) is unknown. In contrast, the time lag of detecting the same sound at Rec 2 (i.e., time delay in detecting deer sound at Rec 2 after detecting it at Rec 3) can be calculated from sound recordings (hereafter, time lag $T_{2}$, as shown in Figure 3a). Similarly, time lag $T_{1}$ represents the time difference between detecting the same deer sound at Rec 3 and Rec 1 (Figure 3a). The distance ($D_{1}$) corresponding to the time lag $T_{1}$ shown in Figure 3a can be calculated as:

(1) $$D_{1}=T_{1}\times V$$

where V represents the speed of sound in air, which depends on the air temperature. For instance, the speed of sound in dry air is 331.20 and 343 m s^−1^ at 0 and 20 °C, respectively. Similarly, the distance $D_{2}$ (Figure 3a) corresponding to the time lag *T*_2_ is calculated as *D*_2_ *= T*_2_ *× V*. Thus, the only information that can be estimated from the sound recordings are time lags $T_{1}$ and $\;T_{2},\;$ and their corresponding distances $D_{1}$ and $D_{2}$.

The estimation of the position of the sound source ($Lat_{0},\;Lon_{0}$) relied on a trial-and-error method. A grid with a tolerance of 0.5 m along both the *x* and *y* axes (Figure 3b) was used to determine the position of the sound source. The grid should provide adequate coverage for detection of sound at all three recorders. Consequently, the distance between any recorder and the grid edge (hereafter called edge distance) was set to be at least 750 m (Figure 3b). In the trial-and-error method, each of the grid’s intersection points was investigated assuming that the deer emitted the sound at that point (e.g., the blue point in Figure 3b). As the coordinates of the intersection points along with three-recorders were known, the distances between investigated point and each of the three-recorders can be calculated (i.e, $D_{inv\_1}^{*},\;D_{inv\_2}^{*}$ and $D_{inv\_3}^{*}$) as shown in Figure 3b. The calculated distances enabled us to identify the nearest recorder from the investigated point, and calculate the distances corresponding to the time lag in the other two-recorders (i.e., $D_{1}^{*},\;D_{2}^{*}$, shown in Figure 3c). The root mean square error (RMSE) was then calculated between the distances corresponding to the time lag of deer calls recorded at Rec 1 and Rec 2 (i.e., $D_{1}$ and $D_{2}$), and time lag distances from the point being investigated (i.e., $D_{1}^{*}$ and $D_{2}^{*}$). By repeating these calculations at all the grid intersection points, the point with the lowest RMSE was extracted. The point with the lowest RMSE represents the retrieved deer position, which matches or is very close to the actual deer position.

The following steps explain the details of the calculations required at each intersection point of the grid, to determine the intersection point nearest to the deer position.

Step (1): For each intersection point of the grid, loop the following steps to be executed. Let us use the blue intersection point shown in Figure 3b,c as an example, with the following steps.

Step (2): Calculate the distances between the point under investigation, and each of the three recorders. The coordinates of the point being investigated ($Lat_{0}^{*},\;Lon_{0}^{*}$) are known, as it is a grid intersection point. Applying the Pythagoras’ theorem on a geographic projection [36], the distance between any two points with known coordinates can be calculated as follows:

(2) $$d=R_{earth}\times \sqrt{x^{2}+y^{2}}$$

(3) $$x=\;\left(Lon_{II}-\;Lon_{I}\right)\times \left(\frac{\pi }{180}\right)\times \cos\left(\frac{Lat_{II}+\;Lat_{I}}{2.0}\times \frac{\pi }{180}\;\right)$$

(4) $$y=\;\left(Lat_{II}+\;Lat_{I}\right)\;\times \left(\frac{\pi }{180}\right)$$

where $R_{earth}$ refers to the mean radius of the earth (6,371,000 m), ($Lat_{I},\;Lon_{I}$) are the coordinates of the first point (I), and ($Lat_{II},\;Lon_{II}$) are the coordinates of the second point (II). The distance from the point under investigation to each of the three recorders (i.e., $D_{inv\_1}^{*},\;D_{inv\_2}^{*},\;and\;D_{inv\_3}^{*}$, as shown in Figure 3b) are calculated using Equations (2)–(4).

Step (3): Determine the minimum distance (hereafter, $D_{min}^{*}$) among the three distances (i.e., $D_{inv\_1}^{*},\;D_{inv\_2}^{*}\;and\;D_{inv\_3}^{*}$) calculated in step (2). This is to determine which recorder is the nearest to the point being investigated. For the point investigated in this example, Rec 3 is the nearest recorder, and $D_{\;min}^{*}$ is equal to $D_{inv\_3}^{*}$.

Step (4): Check if the recorder nearest to the point being investigated is the same recorder that is nearest to the source of the deer call. If yes, the rest of the steps are executed. If not, the loop should terminate, and the next intersection point of the grid should be checked. This condition was set to reduce the computational time. For the point investigated in this example, the nearest recorder is Rec 3, which is the same recorder nearest to the sound source (i.e., Rec 3 was the first recorder to detect the deer sound).

Step (5): Calculate the distances corresponding to the time lag at the other two recorders. For the point under investigation, shown in Figure 3b,c, Rec 1 and Rec 2 are far from the investigating point compared to Rec 3. Thus, the distance $D_{1}^{*}\;$(Figure 3c) is the corresponding time lag distance of Rec 1, which can be calculated by subtracting the distance $D_{\;min}^{*}$ calculated in Step (3) from the distance $D_{inv\_1}^{*}$ calculated in Step (2) ($D_{1}^{*}=D_{inv\_1}^{*}-D_{\;min}^{*}$). Similarly, the distance corresponding to the time lag of Rec 2 can be calculated ($D_{2}^{*}=D_{inv\_2}^{*}-D_{\;min}^{*}$), as shown in Figure 3c.

Step (6): The RMSE between the time lag distances corresponding to the point under investigation (i.e.,$\;D_{1}^{*}$ and $D_{2}^{*}$), and the distances calculated based on the deer sound (i.e., $D_{1}$ and $D_{2}$) is calculated as [37]:

(5) $$\mathrm{RMSE}=\;\sqrt{\frac{{\left[\left(D_{1}^{*}-D_{1}\right)+\left(D_{1}^{*}-D_{2}\right)\right]}^{2}}{2}}$$

Step (7): The above-mentioned procedure should be performed for all the intersection points of the grid (Figure 3b), as explained for the blue intersection point. The point that provides the minimum RMSE represents the position of the deer.

In short, the proposed technique relies on the time lag among three recorders and a trial-and-error method to estimate the emitted sound position. The proposed technique uses three recorders in a triangle layout; however, more recorders with different architecture could be used. The influence of wind speed and relative humidity on sound propagation was not considered in this study.

### 2.3. Development of Sound Recording System

**Figure 4:**
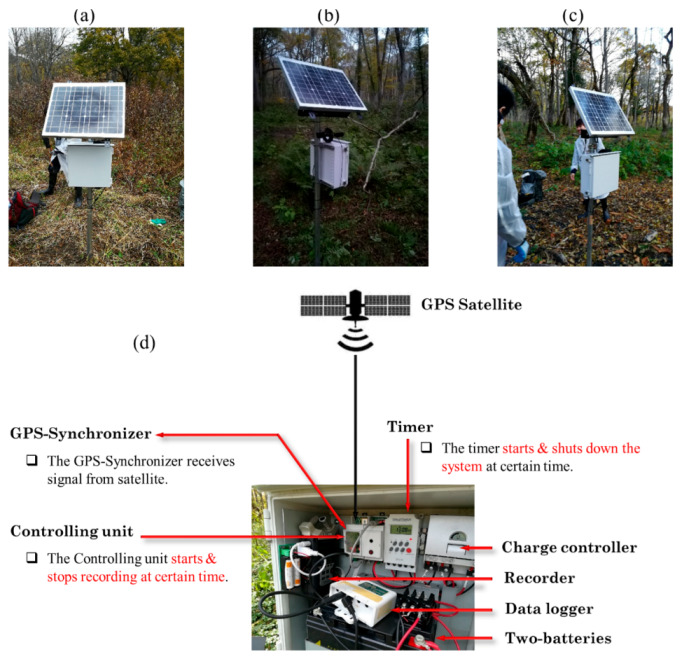
Three sound recorders and solar panels in Oze marshland. (**a**) Recorder 1 (Rec 1), (**b**) Recorder 2 (Rec 2), (**c**) Recorder 3 (Rec 3), and (**d**) components of the sound recording system.

A sound recording system was designed to meet the requirements of our proposed technique. The sound data were captured using an external microphone (i.e., AT8538 Power Module, Audio-Technica, Tokyo, Japan) attached to a Zoom H4n Pro digital recorder (Zoom North America Inc., Hauppauge, NY, USA), with a sampling rate of 44.1 kHz. The acoustic data were saved into an SD memory card in MP3 format, with a 32-bit amplitude resolution. The external microphone was installed approximately 1.5 m above the ground level and pointed towards the region being investigated, as clearly shown in Figure 4b. Solar panels were used to supply power to the whole system including recorders (Figure 4a–c). The latitude and longitude information were obtained from a Garmin eTrex 20 GPS receiver (Garmin International Incorporated Company, Olathe, KS, USA).

There were two main challenges during the collection of sound data. The first challenge was the time drift problem with the sound recorders (i.e., the clock of each recorder does not run at the same rate as the reference clock). For instance, even if the three recorders are manually synchronized at a certain time, they will show different times after a while (e.g., one day). As the proposed technique mainly relies on estimating the time lag among the three recorders, the three recorders should start collecting data at exactly the same time. Because of the time drift problem of the recorders, it was difficult to use the internal clock of the recorder to start sound recording at the same time (i.e., synchronization process). Consequently, a unique sound recording system was developed which consists of a GPS synchronizer, a controlling unit, and a data logger, as shown in Figure 4d. The GPS synchronizer was responsible for receiving accurate time information from GPS satellites every hour. This precise time information was transferred to the controlling unit. The controlling unit started/stopped collecting sound data every hour. The GPS synchronizer also passed a signal to the data logger showing the synchronization status (i.e., whether receiving a signal from GPS satellites or not).

The other main challenge was the power supply in the forest. The amount of sunlight that reaches the solar panel was insufficient in some areas due to the dense tree cover. Consequently, two solar panels (Figure 4b) were used in areas with dense tree cover. On the other hand, the ideal time for recording deer calls is nighttime because that is when the deer are active [38]. Accordingly, a timer device (Figure 4d) was added to stop the system during the daytime (i.e., between 4:00 and 16:00) to save power.

### 2.4. Distance between Sound Recording Units

The distance between the sound recording units is a critical factor for our proposed technique because the deer sound should be recorded by three recorders. In addition, it is important to know the maximum distance at which the deer sound is detectable in Oze, to reduce the total number of devices. The detectable range for deer sounds may vary based on many conditions such as tree density, and recorder sensitivity. For instance, Enari et al. [2] observed that the detection range for passive acoustic monitoring was 140 m in defoliated forests, for deer calls during the rutting season. Masato Minami [31] reported that there are two types of male deer voices in the rutting season; the maximum distances at which these two voices were detectable were 400 and 700 m.

We also conducted an experiment to check the maximum distance at which sound could be detected in Oze. During the experiment, sounds from deer whistles were created at different distances from Rec 1, up to 761 m. Table 2 shows that the sound could be detected within 400 m. The deer whistle was hardly audible at 545 m, and the sound was not detected at all at 761 m. Consequently, the distance between sound recording units was adjusted to be within 450 m, as shown in Figure 2d. The Garmin eTrex GPS receiver (Garmin Ltd., Olathe, KS, USA) was used to determine the position information (i.e., latitude and longitude) of the emitted sound from deer whistle. Praat software (University of Amsterdam, Amsterdam, The Netherlands) was used to analyze the recorded sounds [39].

## 3. Results and Discussion

### 3.1. Validation of the UTokyo Experiment

**Figure 5:**
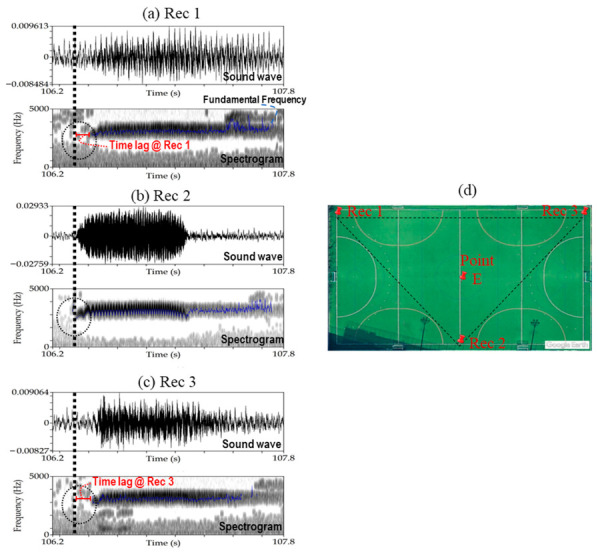
Sound editor windows of Praat software show the sound waves and spectrogram of a recorded sound at (**a**) Rec 1, (**b**) Rec 2, and (**c**) Rec 3 in UTokyo experiment. The recorded sound at the three recorders was emitted from a whistle located at point E whose position is shown in (**d**).

The UTokyo experiment was conducted on 29 November 2017. In the UTokyo experiment, the microphones of the three recorders were installed facing the center of the playground, as shown in Figure 1b. A deer whistle was used to create a sound at five different points in the playground (i.e., A–E, shown in Figure 1b). The measured positions (Table 1) of the five validation points were compared with the positions derived from the proposed technique. The grid size of the trial-and-error method was 1.6 km × 1.6 km, to cover the playground and recorders. The sound editor windows of Praat (Figure 5a–c) illustrate the characteristics of the sound recorded by the three recorders when the deer whistle was used at point E (Figure 5d). The sound recordings were monaural (i.e., one channel) as one external microphone was connected to each recorder (Figure 5a–c). The blue curves shown inside the spectrograms represent the pitch (i.e., the fundamental frequency of sound waves). The pitch curves are one of the factors that can be used to determine the time when the deer sound was first detected in each recorder. The pitch range (i.e., the range in which the fundamental frequency analysis is carried out) is an important setting for pitch analysis and changes based on the target sound. For example, the pitch range for a male voice ranges from 75 to 300 Hz, whereas it is between 100 and 500 Hz for a female voice. By testing different pitch ranges for both the UTokyo experiment (i.e., deer whistle sound) and Oze (i.e., real deer sound), the lower and upper limits of the pitch range were set at approximately 500 and 5000 Hz, respectively.

This paragraph explains the determination of time lag among the three recorders using the measurements carried out at point E of the UTokyo experiment. The whistle sound was emitted at point E, and the sound was first detected at Rec 2 (i.e., the nearest recorder from point E, as shown in Figure 5d) at time 106.316 (i.e., the relative time measured with respect to the starting time of the recordings). The same sound was later detected at Rec 1 and Rec 3 at 106.416 and 106.417, respectively. The time lag for both Rec 1 and Rec 3 from Rec 2 (i.e., the first recorder that detected the sound) was calculated. The time lag at Rec 1 was 0.100 s (106.416–106.316, as shown in Figure 5a), and the time lag at Rec 3 was 0.101 s (106.417–106.316, as shown in Figure 5c). The mean air temperature on 29 November 2017 was 18 °C, and the speed of sound was assumed to be 340 m s^−1^. The Noise Reduction tool of Audacity software [40] was used to reduce the background noise (e.g., rustling leaves and wind noises) in sound recordings, and thus improving the recorded sounds’ quality and facilitating deer calls’ extraction. Noise reduction is a two-step procedure. In the first step, the user provides a sample noise (at least 0.05 s at a sample rate of 44,100 Hz) to train Audacity. Then, Noise Reduction is applied to the entire sound recording [41].

**Figure 6:**
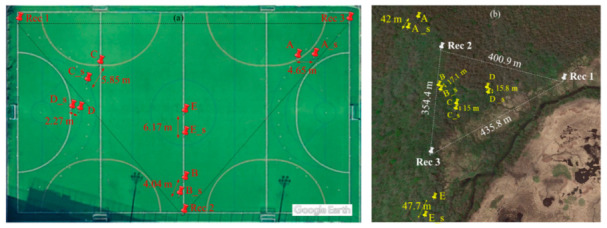
Google Earth images illustrate the positions of the validation points at (**a**) UTokyo experiment and (**b**) Oze marshland. The letters (i.e., A–E) indicate the reference positions of deer whistle and letters with “_s” represent the retrieved positions using the proposed technique. The distance between the reference and retrieve positions represents the retrieval error using the proposed technique.

Figure 6a illustrates the positions of the five reference points (A–E) as well as the retrieved points (A_s, B_s, C_s, D_s, and E_s) from the proposed technique, from the UTokyo experiment. The proposed technique introduced a mean error and RMSE of 4.60 and 4.80 m, respectively. In general, these retrieval accuracies are acceptable for determining deer positions to estimate their population density in Oze.

**Figure 7:**
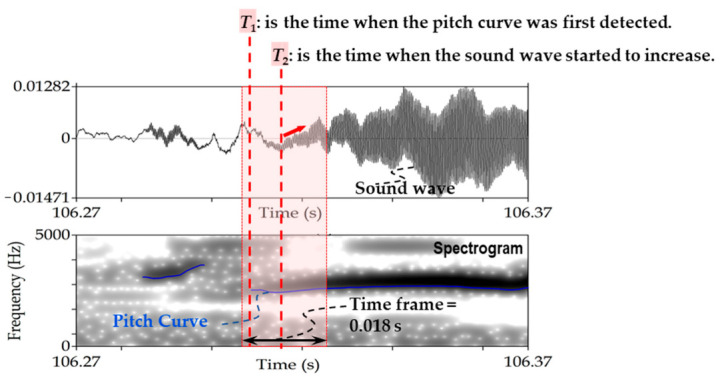
The sound waves and spectrogram at Rec 2 of the UTokyo experiment for detecting the position of Point E (Figure 1b). The highlighted part represents time frame of 0.018 s.

The main factor that can increase error in the retrieved position is the difficulty in manually determining the starting time of deer calls. For instance, the maximum error between the reference and retrieved position was 6.17 m for point E, as shown in Figure 6a. This distance error means that the time error was 0.018 s (i.e., 6.17 m/340 m s^−1^). The time error of 0.018 s is mainly due to lack of human precision to manually determine the time at which the sound was first detected at each recorder. Figure 7 illustrates the sound waves and spectrogram of Rec 2 at time 106.316. The highlighted part in Figure 7 represents the time frame of 0.018 s. Figure 7 emphasizes the challenge of determining the initial detection time for sound within 0.018 s using sound waves and pitch curve, as the time at which the magnitude of sound waves started to increase (i.e., *T*_2_ in Figure 7) was different from the time at which the pitch curve was detected from sound (i.e., *T*_1_ in Figure 7).

### 3.2. Validation of the Oze Marshland Experiment

The validation experiment was conducted in Oze National Park on 14 September 2018. During the validation experiment, the three recorders (Rec 1–3) were used to recorded deer calls emitted from sound whistle at five reference validation points (A–E, shown in Figure 6b). Three out of the five validation points (B–D, hereafter called inner points) were located within the triangle created by the three recorders, whereas the other two points (A and E, hereafter called outer points) were located outside the triangle, as shown in Figure 6b. The latitude and longitude of the five validation points were determined using the Garmin eTrex GPS receiver, and are listed in Table 1. The Noise Reduction tool (Audacity software) was not used in the case of the Oze marshland experiment because of the presence of many sources of irregular background noise. Instead, the sound recordings were amplified to increase the sound volume using the Amplify tool of Audacity software. The grid size for the trial-and-error method in the Oze marshland experiment, and for tracking deer calls in Oze (Section 3.3) was 3 km × 3 km.

**Figure 8:**
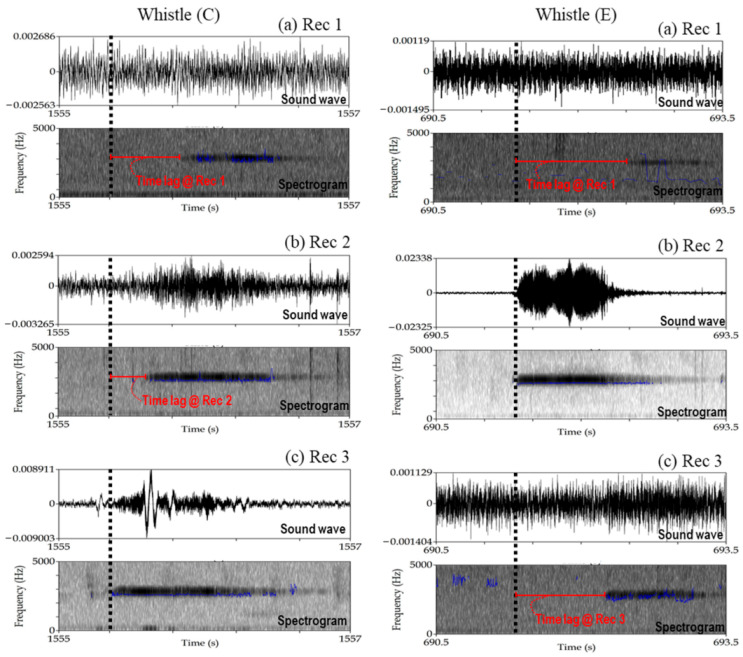
Sound waves and spectrogram of sound recordings from whistle C and whistle E at (**a**) Rec 1, (**b**) Rec 2, and (**c**) Rec 3, during the validation of the Oze Marshland Experiment. The locations of both whistles are shown in Figure 6b. Images were generated using Praat software.

Figure 6b illustrates the positions of deer whistle at five reference validation locations (A–E, Table 1) as well as the retrieved locations (A_s, B_s, C_s, D_s, and E_s) from the proposed technique. The retrieval accuracy of the inner points (i.e., points B–D, Figure 6b) introduced lower error compared with the outer points (i.e., points A and E, Figure 6b), with a mean error of 15.97 and 44.85 m for the inner and outer points, respectively. The reason for the higher error at the outer points may be related to their distant location from the three microphones. For example, Figure 8 shows a comparison between the inner point (point C) and outer point (point E). At point C, the distance to all recorders was less than 400 m and the sound intensity for all recorders was high. In contrast, the distance between point E and Rec 1 (i.e., the farthest recorder from point E among the three recorders) was 495 m, and the sound intensity at Rec 1 was very low, which caused an error in detecting the starting time of the whistle sound at Rec 1. Additionally, increasing the distance between the sound source and the sound recordings along with the presence of other environmental features (e.g., high density of trees or altitude difference) attenuate sound recordings and thereby diminishing the retrieval accuracy, which is consistent with the findings of Castro et al. [42] and Priyadarshani et al. [43]. This conclusion matches our inference in Section 2.4, that the probability of sound detection decreases from approximately 500 m.

### 3.3. Tracking Deer Calls in Oze

Sound recordings of real deer calls in Oze were collected between 8 October and 26 October 2018. October is the rutting season (breeding season), in which the males increase their call range and frequency to attract females. The determination of deer position in an actual deer environment (i.e., Oze) was conducted using four recorders, as shown in Figure 2d (this includes the three recorders used during the Oze marshland validation experiment), in which the distance between recorders remained within 450 m. In order to demonstrate the importance of detecting deer positions, sound recordings on 13 October 2018, between 18:00 and 20:00 (i.e., two-hours) were processed. There were 72 deer calls during the two hours, with an average and standard deviation of 6 calls and 3.59 calls for the 10 min interval, respectively. The animation for the accumulation of deer calls over the two hours considering 30 s intervals is available as Supplementary Material.

**Figure 9:**
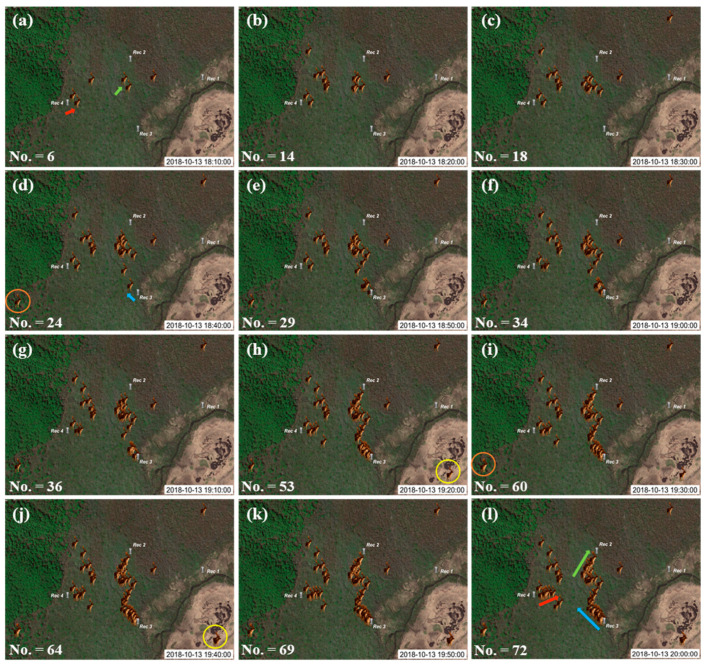
Images show the accumulation of deer call positions from starting time (8:00:00 on 13 October 2018) at 10 min interval for 2 h, as shown in (**a**–**l**). Deer symbols represent the position of deer calls retrieved by the proposed technique. Yellow and orange circles highlight the occurrence of deer calls from the same deer at two different times. The green, red, and blue arrows highlight the repetition and potential path of the same deer with multiple calls. The No. in each figure represents the total accumulated deer calls from the starting time (18:00 on 13 October 2018).

Figure 9 shows the accumulation of deer calls from the start time (i.e., 18:00) to the time posted in each image, over two-hours, and illustrates the changes within a 10 min frame. For instance, there were six deer calls from 18:00 to 18:10 (Figure 9a) increased to 14 deer calls by 18:20 (Figure 9b). The orange circle in Figure 9d highlights the first call of a deer. After 50 min, the same deer made another call, as shown in Figure 9i. Similarly, the yellow circles in Figure 9h,i highlight the first and second calls from the same deer, respectively. The red arrow in Figure 9a highlights the first call from a deer, whereas the red arrow in Figure 9l indicates multiple calls from mostly the same deer. The main reason for assuming that these multiple calls are from the same deer is that the male deer is a territorial animal (i.e., defends a certain area from other males), particularly during the rutting season (from late September to early March) [8]. Consequently, there should be a distance between two males, and if the sound comes from adjacent positions, they are likely to be from the same male. The green arrow in Figure 9a illustrates the first call from a deer. By checking the variation of this deer call along the 12 images, the movement of this deer toward Rec 2 can be clearly seen as highlighted in Figure 9l. Likewise, the blue arrows (Figure 9d,l) and red arrows (Figure 9a,l) clearly show the start and subsequent calls, respectively, from most likely the same deer.

**Figure 10:**
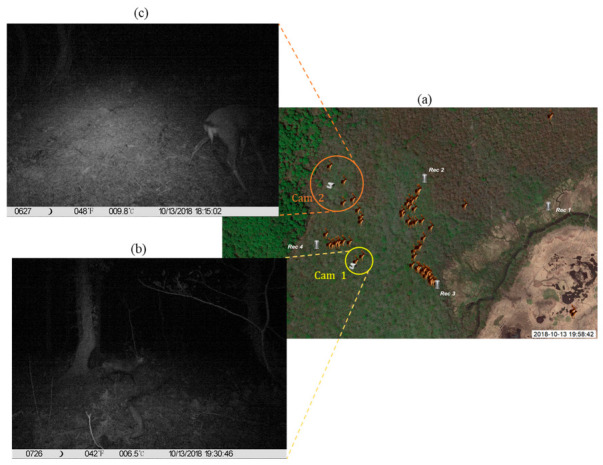
(**a**) Shows the deer call positions retrieved between 18:00 to 20:00 on 13 October 2018 using the proposed technique, along with locations of two camera traps. The deer captured by camera traps 1 and 2 are shown in (**b**,**c**), respectively.

Additionally, there are two camera traps located within our validation site, as shown in Figure 10a. Two deer call positions were retrieved using the proposed technique at 19:11:00 and 19:58:46 (the yellow circle in Figure 10a), located within 25 m from camera trap 1. Camera trap 1 captured a male deer at 19:30:46 (Figure 10b). The positions of four deer calls retrieved using the proposed technique were within 50 m from camera trap 2 between 18:11:26 and 18:53:45 (the orange circle in Figure 10a). Camera trap 2 captured male deer at 18:15:02 (Figure 10c). As both images of the camera traps are for male deer who are territorial animals, we deduce that the deer positions retrieved using our technique correspond to the deer captured by the two camera traps. Further research using marked individuals with tracking devices combined with sound recordings and camera traps are needed to confirm this speculation.

These outcomes underline the significance of the proposed technique for detecting repetitive calls from the same deer and excluding them, when using sound recordings for deer counting, in addition to observing deer movement by determining the position of deer sounds. Accordingly, the proposed method can be a basis for sound-based techniques for counting deer and other vocal animals in Oze and other regions.

## 4. Conclusions and Recommendations

In this study, a new technique to detect deer position (i.e., latitude and longitude) from sound recordings at three recorders was introduced. In the proposed technique, the sound is recorded by three stationary recorders that receive the same sound at three different times based on the distance of the sound source from the three recorders. Then, the time lag in detecting the same sound among the three recorders is estimated. A sound recording system was also developed to receive accurate time information from GPS satellites to overcome the time drift problem of the sound recorders (i.e., the clock of each recorder does not run at exactly the same rate as the reference clock used); therefore, the time lag among the three recorders was accurately estimated. The distance between sound recording units along with the presence of obstacles (e.g., high density of trees) influence sound recordings and hence the retrieval accuracy of the proposed technique. The retrieval accuracy’s mean error of the experimental and natural environment was 4.60 and 15.97 m, respectively. Thus, the developed technique is most suitable for open habitat and difficult-to-access terrain (e.g., muddy wetlands) with sparse tree cover and relatively flat terrain. The proposed technique tracks the movement of deer and can be applied to other vocal animals, and therefore monitor ecological changes. Hence, the technique is the first step toward designing an automated system for estimating deer or other vocal animals’ population using sound recordings, as the position information can be used to exclude repetitive calls from the same animal. The proposed technique was validated using a deer whistle and camera traps. It is highly recommended to validate the proposed technique using marked individuals with tracking devices (e.g., GPS collars) in future applications, particularly when applying the technique with other vocal species.

## Figures and Tables

**Table 1:** Coordinates of recorders (Rec 1–4) and validation points (A–E) where deer calls were emitted using a deer whistle at University of Tokyo experiment (Figure 1b) and Oze marshland (Figure 2d).

	UTokyo Experiment Site			Oze National Park	
	Lat	Long		Lat	Long
Rec1	35°39′38.12″ N	139°40′54.13″ E	Rec1	36°56′31.91″ N	139°13′38.43″ E
Rec2	35°39′36.19″ N	139°40′55.57″ E	Rec2	36°56′34.92″ N	139°13′22.63″ E
Rec3	35°39′37.63″ N	139°40′57.72″ E	Rec3	36°56′23.49″ N	139°13′24.27″ E
A	35°39′37.38″ N	139°40′57.09″ E	Rec4	36°56′27.78″ N	139°13′08.86″ E
B	35°39′36.48″ N	139°40′55.63″ E	A	36°56′38.62″ N	139°13′18.70″ E
C	35°39′37.61″ N	139°40′54.96″ E	B	36°56′30.14″ N	139°13′23.51″ E
D	35°39′37.23″ N	139°40′54.66″ E	C	36°56′28.36″ N	139°13′25.95″ E
E	35°39′37.06″ N	139°40′55.76″ E	D	36°56′30.29″ N	139°13′29.18″ E
			E	36°56′19.48″ N	139°13′25.56″ E

**Table 2:** Sound from deer whistle was created at different distances from Rec 1. Symbols O, ∆ and X represent the audibility status namely adequately detected, barely detected, and cannot be detected, respectively.

Distance from Rec 1	181 m	297 m	423 m	545 m	761 m
Audibility of sound	O	O	O	∆	X

## Supplementary Materials

The following are available online at https://www.mdpi.com/1424-8220/21/3/866/s1, Video S1: The animation for the accumulation of deer calls over two hours.
